# Actinomycosis

**Published:** 1902-12

**Authors:** T. P. Lloyd

**Affiliations:** San Antonio, Texas


### Correspondence.

### Actinomycosis.

SAN ANTONIO, TEXAS, December 3, 1902.

*Texas Medical Journal:*

I have watched with interest the reports of the daily papers regarding a disease endemic in New England among cattle, commonly called foot and mouth disease, and naturally conjecture as to its real nature, etiology and pathology. As yet there has been no detailed report.

Is it possible that this disease is actinomycosis ("lumpy jaw or big jaw"), due to actinomyses or ray fungus first described by Böllinger in 1877 in cattle? Isreal says: "Cattle are infected from vegetables or water, from eating ears of barley or rye when the fungus penetrates through a wound or abrasion." I am unable to find any record of such a disease endemic in this country, nor have I seen a description of the present existing contagion or infection.

In the November issue of the *Johns Hopkins Bulletin* is an interesting article by Dr. William G. Erving, entitled "Actinomycosis Hominis in America," with a report of six cases and a *résumé* of ninety-six cases with a distribution as follows: New York State, 22; Illinois, 15; Massachusetxts, 14; Iowa, 8; Maryland, 7; Pennsylvania, 5; Wisconsin, 4; California, Ohio and Virginia, 3 each; Tennessee, Minnesota and Alabama, 2 each; and Colorado, Kansas, Kentucky, West Virginia and North Carolina, 1 each. Texas is conspicuous for her absence, there having been no cases reported from the Lone Star State.

As nearly all the contagious and infectious diseases common among the lower animals are communicable to man, it is well for us to be constantly on guard, and as physicians we should ever keep a watchful eye towards such conditions "lest we forget."

Actinomycosis can no longer be considered a rare disease, and is almost always fatal. It is characterized by a decided swelling of the lower jaw (hence its name, "lumpy jaw") which breaks down, discharging a peculiar granular pus, containing small sulphur-colored granules containing the ray fungus. This disease may attack the glands of the mouth and neck, the skin, intestines, liver, and pleura; in fact, any organ in the human economy.

It is possible for this disease to be disseminated through milk.

T. P. LLOYD, M. D.

